# The influence of hearing loss and hearing aid use on experienced emotion in everyday listening situations

**DOI:** 10.1177/02692155251326830

**Published:** 2025-03-11

**Authors:** Jack A Holman, Graham Naylor

**Affiliations:** 1 Hearing Sciences (Scottish Section), Mental Health and Clinical Neurosciences, 170718 School of Medicine, University of Nottingham, Glasgow, UK

**Keywords:** Hearing loss, hearing aid, emotion, well-being, affect

## Abstract

**Objective:**

To address the extent to which the emotional experience of everyday listening situations is impacted by hearing loss and hearing aid use.

**Design:**

An exploratory prospective study with an observation arm and an intervention arm utilising smartphone-based ecological momentary assessment over 10 days. A hearing loss group was asked to wear and not wear their hearing aids on alternate days. A normal hearing group completed the surveys without hearing aids.

**Setting:**

Remote study gathering data during daily life.

**Participants:**

Twenty-six experienced hearing aid users with hearing loss and twenty participants with normal hearing thresholds.

**Intervention:**

Rotating hearing aid use on alternate days in the hearing loss group.

**Main measures:**

Participants reported on experienced emotions (valence, arousal and discrete emotion) in listening activities at random points throughout the day, as well as at baseline for related socioemotional variables.

**Results:**

Participants with hearing loss reported similar valence and arousal to the normal hearing group when wearing their hearing aids, but significantly lower when not wearing hearing aids. Wearing (versus not wearing) hearing aids showed a significant beneficial effect on valence and arousal. Discrete emotions were more negative when not wearing hearing aids. End-of-day reports of valence were also more negative. There was no significant effect of listening situation type.

**Conclusions:**

Unaided hearing loss was associated with a negative impact on emotions in listening situations. Hearing aids can restore the emotions experienced in everyday listening situations. The results highlight the importance of socioemotional well-being as a factor and outcome in audiological rehabilitation.

## Introduction

Satisfaction with one's life situation and with health interventions is heavily contingent on the emotional experience of everyday life.^1,2^ Hearing loss makes communication more difficult, which can result in the experience of negative emotions.^3,4^ Due to difficulties in listening situations, and conversations in particular, hearing loss is linked to increased feelings of loneliness and social isolation, as people become disconnected emotionally and physically from those around them.^5,6^ Previous evidence suggests that hearing aids can have a positive emotional impact when measured as part of well-being,^7^ or through depressive symptoms.^8^

Ecological momentary assessment is a term encompassing various techniques which gather data in real-time and in the environment of interest. Ecological momentary assessment is ideally suited and widely used for the measurement of transitory emotional response,^9,10^ particularly the circumplex spatial model of emotion widely used in affective research and in this current study.^11,12^ Results from relevant ecological momentary assessment studies suggest that speech communication situations could be most vulnerable to the effect of hearing loss on emotion and that a positive effect of hearing aid use could exist and be measurable using this methodology.^13,14,15^ There is little quantitative research evidence regarding the impact of hearing loss and hearing aid use on emotions in specific listening situations, particularly using real-time data, and none involving the circumplex model of emotion as used in other populations.

This exploratory study used ecological momentary assessment to address the following research questions:

1. Are there general differences between reported emotional states of people with and without hearing loss, or are there particular listening situations where the groups diverge?
2. In what situations, and for what specific emotions, do hearing aids have systematic positive or negative effects?

## Methods

This research received ethical approval from the West of Scotland Research Ethics Committee (18/WS/0007) and the National Health Service of the United Kingdom R&D (GN18EN094).

### Procedure

Data collection ran from August to December 2021. Although there were very few pandemic-related restrictions on social participation in the UK during this period, the study was conducted remotely (i.e. no visits to the department) because institutional rules did not permit participants to visit our department. The feasibility of this ‘non-contact’ approach has previously been established in our target population.^16^

The protocol began with a baseline session (online) where informed consent was taken, and questionnaires were completed for variables that could theoretically explain some of the variance in results. Following this, researchers contacted the participants with information on how to install, set up and use the app. A start-up survey triggered 10 full days of smartphone-based ecological momentary assessment (11 days including a partial first day if starting after 9 am). Participants with hearing loss were instructed to wear and not wear their hearing aid(s) on alternate days, a technique that has been utilised previously with success.^14^ For these participants, the morning surveys served as a reminder of whether to wear hearing aids or not. As part of the start-up survey participants were instructed on safety while using the app and during the study as well as being informed about using hearing aids when they felt they needed to. They were told never to respond to a survey prompt in a potentially unsafe environment such as while driving or crossing the street. They were also informed not to refrain from using hearing aids if they felt doing so would put them at risk, such as in the aforementioned situations. Participants were informed in the consent forms and in the start-up survey that they could withdraw for any reason at any time.

Ecological momentary assessment survey responses were automatically uploaded to the cloud whenever participants’ smartphones were connected to Wi-Fi. Researchers monitored the data to ensure that participants were not having problems and regularly reached out via email. At the conclusion of the study, participants were contacted by email and recompensed for their efforts. Participants received a £5 voucher for completing the online questionnaires and a further £20 voucher for completing the ecological momentary assessment.

### Participants and recruitment

The study population consisted of two groups. The primary group of interest was adults with hearing loss who used hearing aid(s). The normal hearing group consisted of people with no self-reported hearing loss. Most participants were members of the departmental participant pool. Eight participants with no self-reported hearing loss were recruited through word of mouth via existing participants. The study was described as an online and smartphone study looking at hearing loss, hearing aids and daily life. The protocol for the study (wearing and not wearing hearing aids) meant that blinding would not have been possible in this instance. The participant pool consists of people who have previously taken part in studies and agreed to be contacted for future studies. Participants with hearing loss were invited to the pool after seeking audiological care at Greater Glasgow and Clyde audiology services. Other participants of all hearing abilities have been added over time through recruitment drives (posters, emails, word of mouth). The eligibility criteria for this study were adults aged 18–75 years old who own a smartphone. The wide age range was intended to capture people of different lifestyles, as the affective experience of hearing loss and hearing aids may be different in the older/retired population compared to younger/working/child-raising samples. However, no attempt was made to power this exploratory study to definitively detect such effects. Participants with hearing loss were invited based on previously measured pure tone audiometric thresholds above 25 dB HL in the worse ear. As previous/up-to-date audiometric data was not available for all participants in the normal hearing group, eligibility for the normal hearing group involved responding as having very good, good, or middling hearing during the baseline questionnaire session. We attempted to recruit 60 participants (30 per group) and to control for age and gender across groups. However, difficulties recruiting remotely for the normal hearing group, and drop-outs, meant that fewer datasets than hoped were completed, and there were group differences in baseline characteristics (see Table 1).

**Table 1.** Group demographics.

	Study participants: *N* = 46		
	Hearing loss group	Normal hearing group	*P*-value
*N*	26	20	
Age mean (st dev)	69.2 (6.3)	61.2 (8.2)	<.001
Male	10	3	.16
Female	16	17	
Baseline positive affect (PANAS)	17.6 (3.5)	18.3 (2.9)	.45
Baseline negative affect (PANAS)	11 (4.6)	9.65 (2.6)	.21
SSQ12	3.8 (1.8)	6.95 (2.2)	<.001
SAL	2.4 (0.96)	2.8 (1.3)	.21
SPaRQ SP	49.5 (24.1)	16.5 (22.1)	<.001
SPaRQ SB	42.2 (23.2)	13.3 (19.1)	<.001
HHIA	47.62 (23.8)	15 (22.5)	<.001
4FAHL BE (dBHL)	35 (11.6)	13 (7.3)^a^	<.001
4FAHL WE (dBHL)	50 (16.04)	16 (8.85)^a^	<.001

PANAS: positive and negative affect schedule; SSQ12: speech spatial and qualities scale; SAL: social activity log; SPaRQ SP: social participation restrictions questionnaire social perceptions; SPaRQ SB: social participation restrictions questionnaire social benefit; HHIA: hearing handicap inventory for adults; 4FAHL BE: four frequency average hearing loss better ear; 4FAHL WE: four frequency average hearing loss worse ear. *P*-values from Welch's *t*-test for all variables other than gender where chi-square test was used.

a Excluding those for whom we did not have audiometric data.

The study consisted of 46 participants; 26 participants who had a measured hearing loss and used hearing aid(s) and 20 control participants reporting very good, good or middling hearing. Participants with hearing loss had an average age of 69.2 years (standard deviation 6.3), 16 female (62%). Participants in the normal hearing group (control) had an average age of 61.2 years (standard deviation 8.2), 17 female (85%) (Table 1). All participants with hearing loss had been using hearing aids for at least 5 years.

### Materials

The RealLife Exp smartphone app, which is the user interface of the Lifedata platform^17^ was used for the ecological momentary assessment. Lifedata is a commercially available web-based platform, which allows for the creation and delivery of protocols, and cloud-based data monitoring. Participants all owned a smartphone and used their own phones for the study. Participants were instructed to enable push notifications for the app so that they would be alerted to surveys. This would be by noise, vibration or a combination of both based on preference.

JISC online surveys^18^ were used as the platform for completing baseline questionnaires online. Participants were emailed a unique link through which they could complete the questionnaires. In addition to the questionnaires, the link recorded informed consent and asked for basic information such as a unique identifying number which had been provided by separate email, to ensure that results could be matched to participants.

With the aim of striking a balance between the acquisition of large amounts of valuable data and participant burden/drop-out, the format of the study and the design of the protocol and schedule were carefully considered with reference to similar research.^19^

The start-up survey consisted of confirmatory questions asking for the participants’ unique ID number, age and gender. There was a full description of the survey schedule to support the information previously given by the researchers. There was also a data protection statement and a safety notice informing the participants not to use the phone or interact with the surveys when there was any chance of danger.

Morning surveys started on the second day of the study and were sent each morning at 9.00 until the 11th day of the study inclusive. The questions in the survey are presented in supplementary material 1. Participants had three hours to respond to the survey. In case of no immediate response, reminders were sent at 9.25 and at 9.50 am. Morning surveys recorded valence, outlook and hearing aid plan for the day. The main aim of the morning survey was to ensure that HL participants were aware of the hearing aid schedule and so that they could inform the researchers of any need to deviate.

Six daytime surveys were sent every day from the first day until the 11th day of the study. They were sent between 10.00 and 21.00 at random intervals at least 60 minutes apart. In case of no response to a notification, two reminders were sent at 20-minute intervals. Participants had 45 minutes to respond to each daytime survey before it expired. Daytime surveys recorded situation type, conversation purpose (if applicable), background noise, location, valence, arousal, dominant discrete emotion, and perceived impact of hearing (supplementary material 1). The list of discrete emotions was informed by participant reports from the study by Holman et al. (2022), and completed following scrutiny of relevant literature, to provide a broad and balanced spread of options, eight positive and eight negative.

The end-of-day survey was sent at 21.00 for the duration of the study, starting on the first day. Participants had four hours to respond to the survey. In case of no response, two reminders were sent at 30-minute intervals. The end-of-day survey recorded across-the-day valence, perceived impact of hearing, social connectedness, pleasantness of social interactions, energy levels and adherence to the hearing aid schedule (supplementary material 1). Several of these variables were analysed but are not described in this manuscript for conciseness.

The baseline questionnaires were the Hearing Handicap Inventory for Adults^20^ for the perceived emotional and social/situational consequences of hearing loss, the Social Activity Log^21^ for the level of social activity, the Positive and Negative Affect Schedule^22,23^ for trait affect, the Speech Spatial and Qualities of Hearing Scale for multidimensional hearing ability,^24^ and the Social Participation Restrictions Questionnaire.^25^ To account for varying use of hearing aids amongst participants, all baseline questionnaires were requested to be answered ‘as you are in your everyday life’. In addition, we asked a single question regarding self-reported hearing loss: ‘How is your hearing without aids? Very good, good, middling, poor, very poor’ (5–1). This was partly to ensure that participants in the normal hearing group didn’t consider themselves to have poor or very poor hearing. If this was the case the participants would be excluded from analysis of the normal hearing group. More details on baseline questionnaires (including validity and reliability information) can be found in supplementary material 2.

### Data analysis

Statistical analyses were carried out using R version 4.1.1.^26^ For analysis of descriptive statistics, *t*-tests were conducted where appropriate. Levene's test was used to assess homogeneity of variance and the Shapiro-Wilk test was used to assess normality. Scatterplots and bar charts were used to visually inspect the data before other statistical analyses were conducted. End-of-day survey responses were analysed using simple linear mixed effects models, controlling for age and gender. For analysis of discrete emotion responses, the proportion of positive and negative discrete responses was calculated for each participant for hearing aid and non-hearing aid conditions, followed by analysis using a paired Wilcoxon signed-rank test. This was preferred because the total number of completed surveys was likely to be different between groups and between hearing aid conditions. Multilevel modelling was used to assess the impact of hearing loss and hearing aid use on momentary reports of valence and arousal. This was analysed using the nlme package.^27^ Multilevel modelling is ideally suited to ecological momentary assessment data which often has missing data and violates the assumptions of independence of observations and errors. The hierarchy in this study was survey-level data at the first level, day of the study (1–10) at the second level and participant-level data at the third level. Situation type (conversation = 0, non-conversation = 1) and location (home = 0, outside home = 1) were recoded. A separate model was built for valence and arousal for each comparison of the three conditions (hearing loss group wearing hearing aids, hearing loss group not wearing hearing aids and normal hearing group), resulting in six models in total from daytime survey data. Situational and socioemotional predictor variables were included to assess whether these account for variance in the reported emotional states. The models were created starting with a basic model followed by the introduction of an intercept-only model, a random intercept model, adding main fixed effects, introducing random slopes and adding predictor variables. At each step, an assessment was made using analysis of variance as to whether or not the change or addition of variables improved the model. Variables were added individually, with those not improving the model removed. Demographic variables age and gender were added to models first. Situation type (conversation or no conversation) was then added to check whether situation type or the interaction of situation type and group (hearing loss or hearing aid use) was significantly related to valence or arousal. The other situational and socioemotional variables of noise, location, day of study, hearing handicap, social participation restrictions, social activity level, baseline affect and multidimensional hearing ability were then added to investigate whether they could improve the model.

Participants with a daytime survey response rate (number of surveys completed/number of surveys issued) of less than 60% were to be removed from the analysis, although in the event, all participants surpassed this threshold. Age and baseline questionnaires were grand mean centred across all study participants. Survey-specific data (location, situation type and noisiness) were person-mean centred.

## Results

### Descriptive results

In the hearing loss group, 1642 surveys were issued in total across both conditions, of which 163 were not responded to, for a combined response rate of 90.1%. In the normal hearing group, there were 1235 surveys in total, 191 not responded to, for a combined response rate of 84.5%. Twenty-three (13 normal hearing, 10 hearing loss) surveys were responded to but not completed and thus were not included in analysis. Participants with hearing loss followed the intended alternating pattern of hearing aid use on 97% (258/266) of the study days. Reasons given for not following the pattern of hearing aid use were a cinema visit, hospital appointment, flu vaccination, attending a family function, high winds when playing golf and three unspecified reasons. Non-compliance with the intended pattern did not impact analysis as hearing aid use was recorded at each daytime survey.

**Figure 1.**
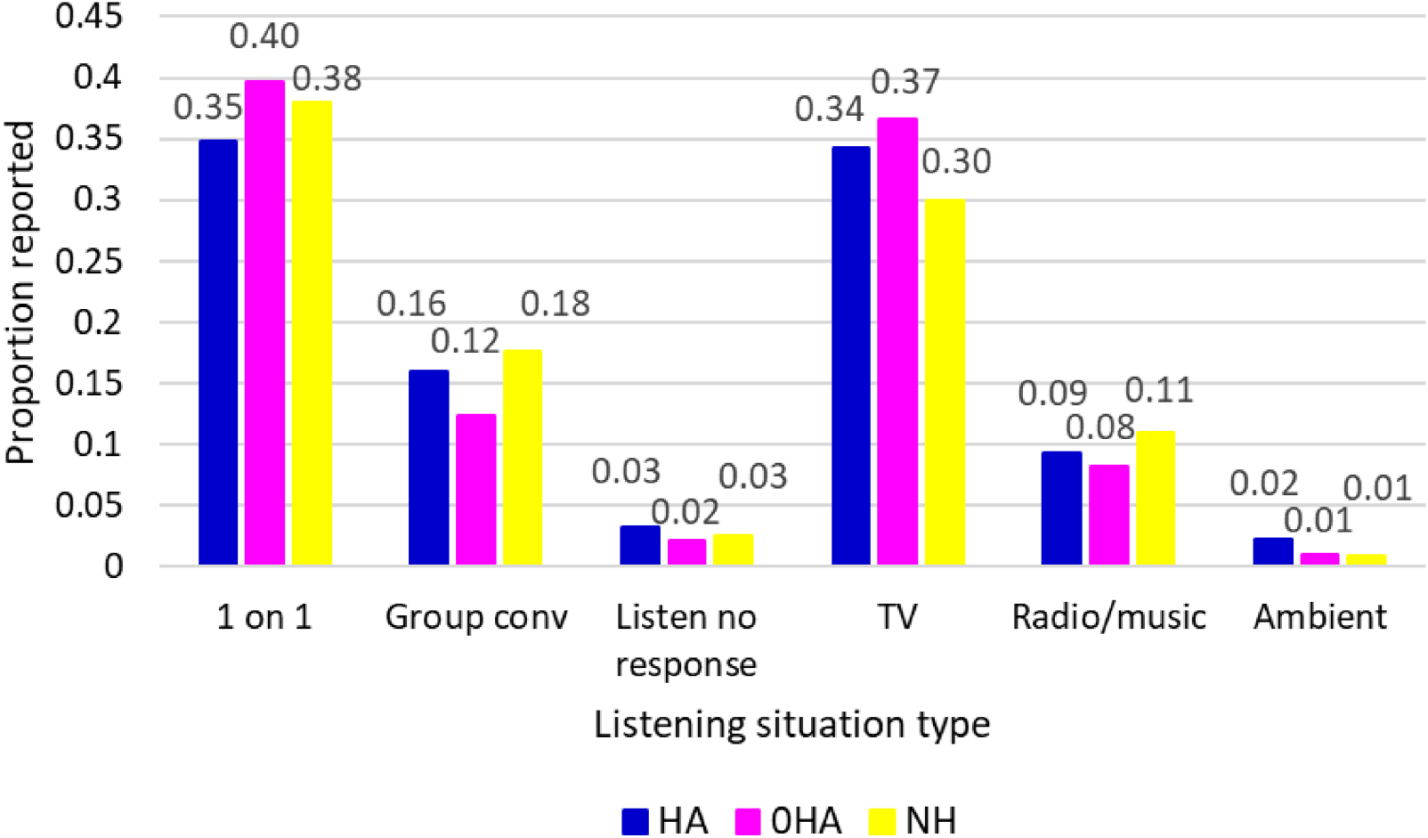
Listening situation type as a proportion of total survey responses per group and aided/unaided conditions. HA: wearing hearing aid; 0HA: not wearing hearing aid; NH: normal hearing.

The distribution of listening situation types reported by participants was broadly similar across groups, and hearing aid versus no-hearing aid subgroups in the case of participants with hearing loss. The least reported (listening to ambient noise) was reported 27 times. Next to that, being in a one-way listening situation, such as listening to a presentation was reported 54 times. One-to-one conversations and listening to television were the most reported listening situations (figure 1).

The hearing loss group was significantly older (mean difference 8 years) than the normal hearing group (*t*(44) = −3.63, *P* = <.001). While a chi-square test for gender differences across groups was not significant (*X*^2^ (1, *N *= 46) = 2.02, *P *> .05), there was a lower proportion of men in the normal hearing group due to a pattern of dropouts for older men in the normal hearing group. There were no significant group differences for positive or negative affect based on baseline questionnaire scores (*t*(44) = 0.77, *P* = >.05; *t*(44) = −1.3, *P* = >.05, respectively). While it was not possible to take audiograms for the participants due to the remote nature of the study, historical audiograms existed for some participants in the normal hearing group and all participants in the hearing loss group. Mean four frequency averages for the hearing loss group were 35 dB HL in the better ear and 50 dB HL in the worse ear. With audiogram dates ranging from 1 year to 6 years prior to the study taking place, the true averages at the time of the study would likely have been higher as hearing loss does not tend to improve and often gets worse with time.

RQ1: Are there general differences between reported emotional states of people with and without hearing loss, or are there particular listening situations where the groups diverge?

### Hearing loss (aided) vs normal hearing

While the group mean valence was slightly higher for the normal hearing group, there was no significant difference between people without hearing loss and people with hearing loss while wearing their hearing aids for valence (*B* = 0.1, *t*(44) = 0.47, *P* > .05) or arousal (*B* = −0.2, *t*(44)= −0.87, *P* > .05). There was no significant effect of listening situation type (conversation or non-conversation) or the interaction between hearing group and listening situation type (which would account for hearing groups experiencing differing valence in different listening situation types) on either valence (*B* = −0.02, *t*(1383)= −0.19, *P* > .05) or arousal (*B* = −0.14, *t*(1383)= −1.24, *P* > .05).

### Hearing loss (unaided) vs normal hearing

When comparing people without hearing loss to those with hearing loss while not wearing hearing aids, there was a significant effect of group on valence (*B* = 0.7, *t*(44) = 2.4, *P* = .022) before including the effect of other situational and socioemotional variables. Adding situation type (conversation versus non-conversation) did not improve the model, and nor did adding the interaction between hearing group and situation type. When comparing people with hearing loss when not wearing hearing aids and people with normal hearing, the addition of hearing handicap (HHIA) to the model resulted in hearing group no longer being significantly related to valence (*B* = 0.5, *t*(41) = 1.64, *P* > .05). Valence was negatively associated with: day of the study (day 1 to 10), (*B* = −0.025, *t*(1339)= −2.8, *P* = .005); noise (*B* = −0.26, *t*(1339)= −6.7, *P* < .001); baseline multidimensional hearing ability (*B* = −0.19, *t*(41)= −2.8, *P* = .008) and baseline hearing handicap (*B* = −0.02, *t*(41)= −3.3, *P* = .0023). Valence increased with baseline social activity level (*B* = 0.33, *t*(41) = 2.9, *P* = .005).

Following a basic model, there was no significant effect of group on arousal (*B* = 0.24, *t*(44) = 0.9, *P* > .05). However, the addition of age and gender significantly improved the model and gender accounted for much of the variance regarding the impact of group on arousal (*B* = −0.7, *t*(41)=−2.5, *P* = .017), as evidenced when looking at hearing group means split by gender (table 2). Adding situation type and the interaction between hearing group and situation type both improved the model, with the interaction between hearing group and situation type being significantly related to arousal (*B* = −0.32, *t*(1339)= −2.6, *P* = .009). This can be interpreted as the normal hearing group reporting higher arousal than the hearing loss group when not wearing hearing aids, and more so in non-conversation situations. Arousal also increased with day of the study (days 1–10), (*B* = 0.03, *t*(1337) = 3.12, *P* = .002) and noise (*B* = 0.11, *t*(1337) = 2.5, *P* = .013). In the final model hearing group was still significantly related to arousal (*B* = 0.8, *t*(41) = 2.64, *P* = .011).

**Table 2.** Mean and standard deviation of momentary valence and arousal for each group by gender and hearing aid status.

	Valence mean (st dev)				Arousal mean (st dev)		
Gender	No hearing aid	Hearing aid	Normal hearing		No hearing aid	Hearing aid	Normal hearing
Male	4.77 (1.23)	5.03 (1.09)	5.37 (1.09)		4.85 (1.08)	4.98 (0.96)	4.98 (1.15)
Female	4.53 (1.53)	5.26 (1.15)	5.28 (1.29)		4.17 (1.35)	4.83 (1.18)	4.65 (1.46)

Note. Range of valence and arousal 1–7.

When comparing end-of-day survey data between the normal hearing group and the hearing loss group on days that they wore their hearing aid(s), there was no significant group difference for the variable emotional valence (*B* = −0.12, *t*(42) = −0.38, *P* = .7).

RQ2: In what situations, and for what specific emotions, do hearing aids have systematic positive or negative effects?

**Figure 2.**
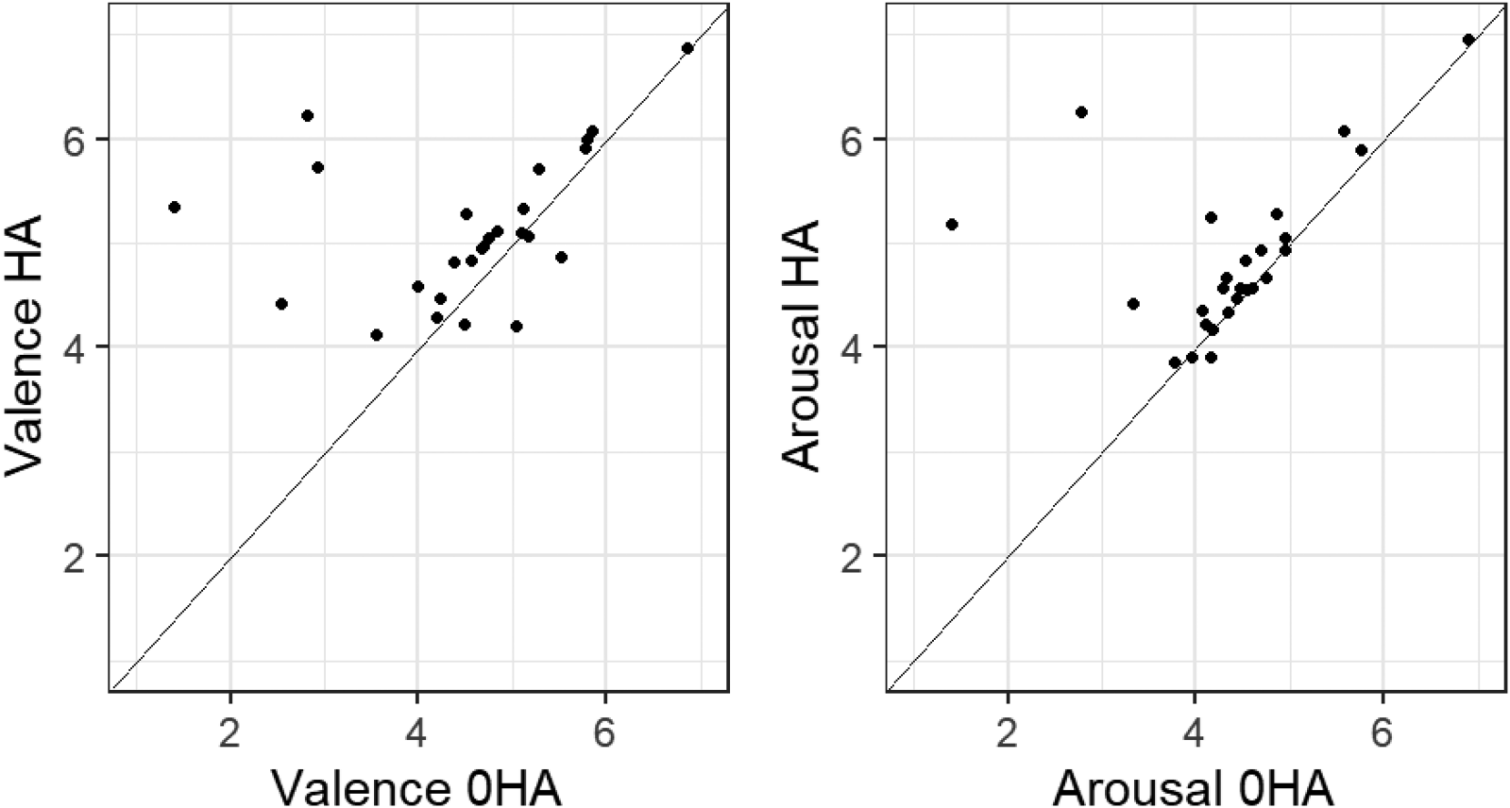
Mean valence and arousal responses of individuals in the hearing loss group aided versus unaided.

As a starting point for investigating the impact of hearing aid use, scatterplots were created for mean valence and arousal for each individual on days they were versus were not wearing hearing aids (Figure 2). There was an improvement in both variables when aided for most, but not all, participants. Those who reported the largest increases in arousal were also among those reporting large increases in valence.

The use of hearing aids was significantly related to higher valence scores (by 0.59 scale points on average but with a wide 95% confidence interval of 0.16–1.03). Adding situation type (conversation versus nonconversation) did not improve the model, and neither did add the interaction between hearing aid use and situation type.

Use of hearing aids was significantly related to higher arousal scores (by 0.45 scale points on average and a 95% confidence interval of 0.083–0.82). Adding situation type did statistically improve the model. However, there was no significant effect of situation type itself (conversation versus non-conversation) on arousal (Table 3). For the end-of-day surveys, there was a significant difference between scores on days where the hearing aid(s) were worn, compared to days when they were not, for the retrospectively reported variable emotional valence (*B* = 0.49, *t*(217) = 3.53, *P* = <.001).

**Table 3.** Effects of predictors on momentary valence and arousal for hearing loss group.

Construct	*B*	SE	T	CI
*VALENCE*				
*CONTROLS*				
HEARING HANDICAP	−0.013	0.005	−2.96**	−0.023, −0.004
SOCIAL ACTIVITY	0.24	0.11	2.12*	0.007, 0.46
*LEVEL 1 VARIABLES*				
BACKGROUND NOISE	−0.26	0.039	−6.67***	−0.33, −0.18
*LEVEL 2 VARIABLES*				
DAY OF THE STUDY	−0.025	0.009	−2.82**	−0.043, −0.008
*LEVEL 3 VARIABLES*				
HEARING AID USE	0.59	0.22	2.7**	0.16, 1.03
AROUSAL				
*CONTROLS*				
POSITIVE AFFECT	0.097	0.035	2.8*	0.025, 0.17
*LEVEL 1 VARIABLES*				
SITUATION TYPE	−0.072	0.054	−1.33	−0.18, 0.033
*LEVEL 2 VARIABLES*				
DAY OF THE STUDY	0.03	0.009	3.5***	0.013, 0.047
*LEVEL 3 VARIABLES*				
HEARING AID USE	0.45	0.19	2.4*	0.083, 0.82

*P < .05 **P < .01 ***P < .001.

Increasing day of the study, increased background noise, and increased hearing handicap were related to decreased valence. Increased social activity level was significantly related to increased valence (Table 3). The day of the study and higher baseline positive affect were related to increased arousal (Table 3).

### Discrete emotions

**Figure 3.**
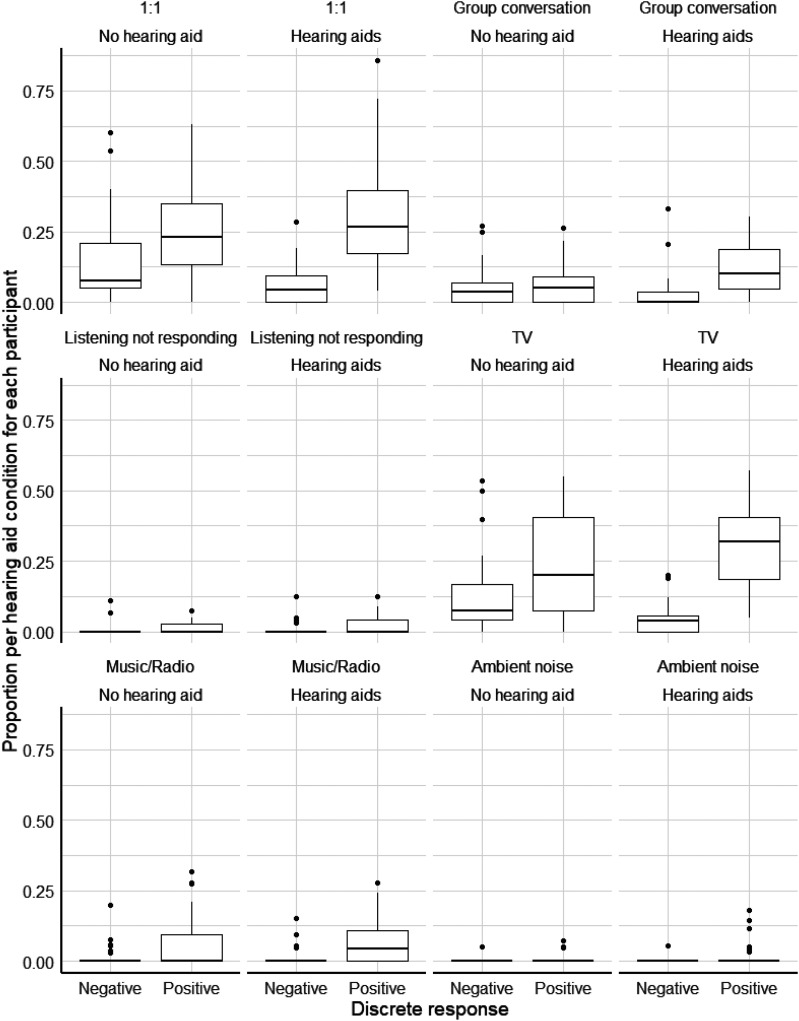
Boxplots of participant total proportion of discrete responses when aided or unaided, grouped by situation type.

Recall that participants could only report one discrete emotion per survey. Tallying across all participants with hearing loss, the balance between positive and negative discrete emotions shifted in the positive direction aided compared to unaided (Figure 3). However, there was also a slightly higher number of total survey reports aided, so for statistical testing, the values were treated as proportions for each participant in each condition. When aided, the proportion of positive discrete emotions was significantly higher (*P* = .006, *r* = .55). Given that the majority of responses corresponded to one-on-one conversations, group conversations and watching television, the increase in positive responses in the hearing aid condition is most evident for these situations (Figure 3). The effect of hearing-aid use on the proportion of positive responses was greatest for one-on-one and group conversation situations. In terms of specific discrete emotions, ‘relaxation’ was the most common response for both aided (33%) and unaided (22%) conditions. Compared to the unaided condition, the largest percentage changes in reported discrete emotions in the aided condition were relaxation (+11%), frustration (−5.6%) and tiredness (−4.7%).

## Discussion

This study found no differences in valence or arousal between the normal hearing group and the hearing loss group when aided. The lack of between-groups differences in affective reports is perhaps surprising given previous evidence that hearing aids are not unanimously popular^28^ and that people often consciously choose when to wear their hearing aids.^4^ Experienced hearing aid users presumably experience some benefit from hearing aids. There are people with hearing loss who qualify for hearing aids but do not use them because they feel they get little benefit, and they wouldn’t have been eligible for this study. The finding that untreated hearing loss negatively impacted emotions in listening situations has been suggested previously via a variety of different methodologies.^29^ We showed a larger effect of hearing loss on valence than arousal. Emotion perception research has shown an effect of hearing loss on valence but not on arousal.^30,31^

Participants in the normal hearing group reported comparatively higher arousal in non-conversational situations, refuting our hypothesis that primarily conversational situations would be where the groups differed. Among the variables related to affective responses, increases in noise and the day of the study were related to decreases in both valence and arousal. The signal-to-noise ratio of natural sounds has been linked to higher ratings of valence and lower ratings of arousal for natural sounds.^32^ Females reported lower valence and arousal than males in every group and condition other than valence in the aided condition. There is mixed evidence of females reporting lower valence and arousal^33^ or an absence of gender effects.^34^

Participants with hearing loss reported significantly higher valence and arousal, as well as more positive discrete emotions, on days they wore hearing aids compared to days on which they did not. This supports the view that hearing aids can have an enriching effect on the emotional experience in listening situations.^4,35^ Given the lack of difference between the hearing loss aided condition and normal hearing group, and the existence of a difference between hearing loss unaided condition and normal hearing group, in this sample, the use of hearing aids appears to have provided a range of affective experience similar to that of people with no hearing loss. This is of course not generalisable to every person with hearing loss given individual differences in the experience of hearing loss and relationships with hearing aids, but it is a potentially important finding for audiological rehabilitation. This is in contrast to emotion perception literature which generally has shown no improvement in emotion identification or affect ratings of stimuli.^36,37^

Higher background noise was associated with lower valence, but not lower arousal. As background noise is by definition not the focus of a listening situation, it makes sense that noise would reduce the positivity of a situation but not directly influence the intensity of the emotional experience. The positive association found between social activity and valence is in keeping with the wider literature. Social activity is beneficial for reports of happiness and social and emotional well-being,^38^ and it can even result in a higher perceived benefit from hearing aid use.^39^ Previous research has identified that certain locations and situations such as work are related to lower self-reported well-being, while our research suggests that all listening situations are equal in terms of affect.^40^ While the concepts of affective experience and well-being are not identical, they are related. We found that hearing aid use was significantly related to arousal only until hearing handicap was introduced. This suggests that the impact of one's hearing loss on daily life may be a stronger determinant of arousal than hearing aid use.

There are several limitations of the study that should be highlighted. At the outset, we aimed to balance the hearing loss and normal hearing groups for age and gender. However, due to several late dropouts from men in particular, the ages and proportion of men in the normal hearing group were lower than in the hearing loss group. As men generally reported higher valence and arousal in this study, the normal hearing group mean values we see here may underestimate the values that would have been obtained, had the group been fully gender balanced. We also recruited experienced hearing aid users, which may neglect to cover people who have hearing loss but are not satisfied with hearing aids. The study took place in 2021, and while there were few pandemic-related restrictions at the time, it is possible that participants were in fewer, and less complex, listening situations than would have been the case under fully normal circumstances. It is possible that the effect of hearing loss would be larger in accordance with the increased frequency and complexity of listening situations in daily life. Given the exploratory nature of the study, we were primarily interested in discovering whether hearing aid use has any impact at all on experienced emotion. Although we also investigated the potential role of a few related variables such as hearing handicap and social activity, there are many other potentially relevant factors such as severity and laterality of hearing loss and aiding, duration of hearing-aid use, age, working status, etc., that were not investigated.

The results of this exploratory study show firstly that ecological momentary assessment can be used to measure emotions in adults with hearing loss via the circumplex model. We found that hearing loss is associated with lower valence and arousal in listening situations in everyday life and that hearing aid use can improve the affective experience for many, but not all, people. This new information on the benefits of hearing aid use is particularly important due to sub-optimal uptake and utilisation rates. The results highlight the importance of socioemotional well-being in auditory rehabilitation and the need for individualised assessment and care. The next step in the research should be to identify whether the beneficial impact of hearing aid use on emotional experience found here for existing hearing-aid users is replicated for people with previously untreated hearing loss following their first-ever hearing-aid fitting, as this could have a substantial clinical impact.

**Clinical messages**

- Hearing loss can negatively impact the emotions experienced in everyday listening situations, which can be measured in real time.
- Hearing aids could improve the emotions experienced in everyday listening situations.
- Affective experience and socioemotional well-being could be an important consideration in clinical consultations and during audiological rehabilitation.

## Supplemental Material

sj-docx-1-cre-10.1177_02692155251326830 - Supplemental material for The influence of hearing loss and hearing aid use on experienced emotion in everyday listening situations Supplemental material, sj-docx-1-cre-10.1177_02692155251326830 for The influence of hearing loss and hearing aid use on experienced emotion in everyday listening situations by Jack A Holman and Graham Naylor in Clinical Rehabilitation

sj-docx-2-cre-10.1177_02692155251326830 - Supplemental material for The influence of hearing loss and hearing aid use on experienced emotion in everyday listening situations Supplemental material, sj-docx-2-cre-10.1177_02692155251326830 for The influence of hearing loss and hearing aid use on experienced emotion in everyday listening situations by Jack A Holman and Graham Naylor in Clinical Rehabilitation
