# Supplementary material for: The influence of hearing loss and hearing aid use on experienced emotion in everyday listening situations
Source: Clin Rehabil. 2025 Mar 11;39(6):770–83. doi: 10.1177/02692155251326830 (PMC12141770; doi:10.1177/02692155251326830)
Supplement: sj-docx-1-cre-10.1177_02692155251326830 - Supplemental material for The influence of hearing loss and hearing aid use on experienced emotion in everyday listening situations [file sj-docx-1-cre-10.1177_02692155251326830.docx]

Supplementary material 1

Morning survey questions

|  | |  | |  | |
| --- | --- | --- | --- | --- | --- |
| Variable | | Question | | *Rating scale* | |
| Emotional valence | | 1. How are you feeling this morning? | | *(slider)* Very negative (1) – very positive (7) | |
| Outlook | | 1. What is your outlook for the day ahead? | | 1. Very apprehensive 2. Somewhat apprehensive 3. Neither excited nor apprehensive 4. Somewhat excited-very excited | |
| Hearing aid plan  (response results in a prompt about whether to wear, or not wear, today) | | 1. Were you wearing your hearing aid(s) yesterday? | | 1. Yes 2. No 3. I do not own hearing aid(s) | |

Daytime survey questions

|  | |  |
| --- | --- | --- |
| Variable | Question | Rating scale |
| Listening | 1. Are you currently, or have you recently, listened to someone or something? | Yes (1) – No (2)  *(Questions 2-9 only fired if the answer to Q1 was Yes)* |
| Listening situation | 2. What was the listening situation? (Coded as 0=Conversation 1=Not a conversastion) | 1. Conversation with one other person 2. Conversation with multiple others 3. Listening to a person speaking without responding 4. TV 5. Radio/music/podcast/audiobook 6. Ambient sounds (e.g. birdsong)   *(Question 2.a. Only fired if the answer to Q2 was (1) or (2)* |
| Conversation purpose | 2.a. What was the purpose of the conversation? | 1. Social 2. Work 3. Transactional (e.g. doctor or shopping) |
| Background noise | 3. What was the background noise level? | 1. Quiet 2. Somewhat noisy 3. Noisy 4. Very noisy |
| Location | 4. Where were you? (Coded as 0=Home 1=Not at home) | 1. Home 2. Work 3. Outdoors 4. Shop 5. Restaurant/café/bar 6. Travelling 7. Other |
| Emotional valence | 5. How positive or negative did you feel? | *(slider)* Very negative (1) – very positive (7) |
| Emotional arousal | 6. How intense was the emotion? | (slider) Very low (1) – very high (7) |
| Discrete emotion | 7. What was the main emotion that you felt during the listening situation? One selection only. | 1. Anxiety 2. Happiness 3. Embarrassment 4. Relaxation 5. Boredom 6. Satisfaction/contentment 7. Tiredness 8. Cheerfulness 9. Sadness 10. Excitement 11. Loneliness 12. Interest 13. Frustration 14. Confidence 15. Irritation 16. Amusement |
| Perceived impact of hearing | 8. To what extent do you think that your ability to hear had an impact on your emotions? | 1. Very negative impact 2. Somewhat negative impact 3. No impact 4. Somewhat positive impact 5. Very positive impact |
| Hearing aid use | 9. Were you wearing your hearing aid(s) during the listening situation? | 1. Yes 2. No 3. I do not own hearing aids |

End-of-day survey questions

|  | |  |
| --- | --- | --- |
| Variable | Question | Scale |
| Emotional valence | How positive or negative have you felt today in general? | (Slider 1-7) Very negative -very positive |
| Perceived impact of hearing | To what extent do you think your ability to hear had an impact on your emotions today? | 1. Very negative impact 2. Somewhat negative impact 3. No impact 4. Somewhat positive impact 5. Very positive impact |
| Social connectedness | How socially connected have you felt today? | 1. Very isolated 2. Somewhat isolated 3. Neither isolated or connected 4. Somewhat connected 5. Very connected |
| Pleasantness of social interactions | How pleasant have your interactions with others been today? | 1. Very unpleasant 2. Somewhat unpleasant 3. Neither pleasant or unpleasant 4. Somewhat pleasant 5. Very pleasant |
| Level of energy | How are you feeling right now in terms of your energy? | 1. Very drained 2. Somewhat drained 3. Neither drained nor energetic 4. Somewhat energetic 5. Very energetic |
| HA use | Did you stick to the hearing aid plan today? (either in all day or out all day) | 1. Yes, I stuck to the plan 2. No I did not stick to the plan 3. I do not own hearing aid(s) |
