# Supplementary material for: The influence of hearing loss and hearing aid use on experienced emotion in everyday listening situations
Source: Clin Rehabil. 2025 Mar 11;39(6):770–83. doi: 10.1177/02692155251326830 (PMC12141770; doi:10.1177/02692155251326830)
Supplement: sj-docx-2-cre-10.1177_02692155251326830 - Supplemental material for The influence of hearing loss and hearing aid use on experienced emotion in everyday listening situations [file sj-docx-2-cre-10.1177_02692155251326830.docx]

Supplementary material 2

*Hearing Handicap Inventory for Adults (HHIA)*

The HHIA is a modification of the Hearing Handicap Inventory for the Elderly (HHIE; Ventry & Weinstein, 1982). It is a tool for quantifying the perceived emotional and social/situational consequences of hearing loss, and is a 25-item self-assessment scale composed of two subscales (emotional and social/situational). Both scales have been shown to have good construct validity, high internal consistency, high test-retest reliability and a low standard error of measurement (Newman and Weinstein, 1988; Newman et al. 1990; Newman et al., 1991).

*Social Activity Log (SAL)*

The SAL (Syrjala et al., 2010) aims to capture the frequency and diversity of social activities outside of daily responsibilities. It consists of 17 items. The first question is “Think about each day of the past seven days and what you did other than working, taking care of your family, or doing necessary shopping. How many days in the past week did you do voluntary social activities?” The remaining items gauge how many days in the past month the person participated in common social activities (e.g. playing cards or games, going to a sporting event, and talking on the phone with friends). Scores range from 0-6. The SAL is reported to have good content and convergent validity, and high internal reliability for the total score but not subscales (Syrjala et al., 2010).

*Positive and Negative Affect Schedule (PANAS)*

Positive affect reflects the extent to which a person feels enthusiastic, active, and alert; whereas negative affect is a general dimension of subjective distress and unpleasurable engagement (Watson et al., 1988). Both scales range from 10-50. The positive and negative affect scales have been shown to have excellent reliability (Ostir et al., 2005), high internal consistency and evident convergent and discriminant validity (Watson et al., 1988).

*Social Participation Restrictions Questionnaire (SPaRQ)*

To assess the psychosocial component of social activity, the SPaRQ (Heffernan et al., 2019) was used. The SPaRQ consists of two subscales, assessing social behaviours and social perceptions. These subscales contain nine and ten questions, respectively, with possible scores on visual analogue scales ranging from 0 (completely disagree) to 10 (completely agree) with rescoring of certain items. The final score range for each subscale is from 0 to 90, and from 0 to 92 respectively, with higher scores indicating greater social participation restriction. The scale has been demonstrated as having high Pearson separation reliability, construct validity and internal consistency (Heffernan et al., 2019).

*Speech, Spatial and Qualities of Hearing scale (SSQ12)*

The SSQ12 (Noble et al. 2013) is designed to measure self-reported hearing ability/disability via twelve items forming three separate subscales. The subscales can be totalled to give an overall score. Each item is scored between 0 and 10, with total scores transformed on a 0-10 scale. The scale has been demonstrated as having robust validity and high reliability in multiple languages (Cildir et al., 2021; Ahlberg et al., 2024).

Ventry IM, Weinstein BE. The hearing handicap inventory for the elderly: a new tool. *Ear Hear* 1982; 3: 128-134.

Newman CW, Weinstein BE. The hearing handicap inventory for the elderly as a measure of hearing aid benefit. *Ear Hear* 1988; 9: 81-85.

Newman CW, Weinstein BE, Jacobson GP, et al. Test-retest reliability of the hearing handicap inventory for adults. *Ear Hear*. 1991; 12: 355-357.

Ostir GV, Smith PM, Smith D, et al. Reliability of the Positive and Negative Affect Schedule (PANAS) in medical rehabilitation. *Clin Rehabil* 2005; 7: 767-769.

Çildir B, Kılıç S, Özkişi B, et al. The Turkish Short Version of the Speech, Spatial, and Qualities of Hearing Scale (SSQ) for Clinical Use: Determining Reliability and Validity for People with and without Hearing Loss on the Basis of SSQ12-A, SSQ12-B, SSQ12-C. *ENT Updates* 2021; 11.

Ahlberg S, Brännström J, Öberg M, et al. An Evaluation of the Psychometric Properties of the Short Form of the Speech, Spatial and Qualities of Hearing Scale in Swedish: Online Versus Paper-and-Pen. *Am J Audiol* 2024; 33: 1176-1183.
